# Antioxidant and Anti-Inflammatory Potential of Polyphenols Contained in Mediterranean Diet in Obesity: Molecular Mechanisms

**DOI:** 10.3390/molecules26040985

**Published:** 2021-02-12

**Authors:** Abdelhafid Nani, Babar Murtaza, Amira Sayed Khan, Naim Akhtar Khan, Aziz Hichami

**Affiliations:** 1 Laboratory of Saharan Natural Resources, African University Ahmed Draia, Adrar 01000, Algeria; 2 Physiologie de la Nutrition & Toxicologie, U1231 INSERM/Université de Bourgogne-Franche Comté (UBFC)/Agro-Sup, 21000 Dijon, France; babarmurtaza87@gmail.com (B.M.); Amira.Khan@u-bourgogne.fr (A.S.K.); naim.khan@u-bourgogne.fr (N.A.K.)

**Keywords:** MedDiet, polyphenols, obesity, oxidative stress, inflammation, AMPK, NF-κB

## Abstract

Nutrition transition can be defined as shifts in food habits, and it is characterized by high-fat (chiefly saturated animal fat), hypercaloric and salty food consumption at the expense of dietary fibers, minerals and vitamins. Western dietary patterns serve as a model for studying the impact of nutrition transition on civilization diseases, such as obesity, which is commonly associated with oxidative stress and inflammation. In fact, reactive oxygen species (ROS) overproduction can be associated with nuclear factor-κB (NF-κB)-mediated inflammation in obesity. NF-κB regulates gene expression of several oxidant-responsive adipokines including tumor necrosis factor-α (TNF-α). Moreover, AMP-activated protein kinase (AMPK), which plays a pivotal role in energy homeostasis and in modulation of metabolic inflammation, can be downregulated by IκB kinase (IKK)-dependent TNF-α activation. On the other hand, adherence to a Mediterranean-style diet is highly encouraged because of its healthy dietary pattern, which includes antioxidant nutraceuticals such as polyphenols. Indeed, hydroxycinnamic derivatives, quercetin, resveratrol, oleuropein and hydroxytyrosol, which are well known for their antioxidant and anti-inflammatory activities, exert anti-obesity proprieties. In this review, we highlight the impact of the most common polyphenols from Mediterranean foods on molecular mechanisms that mediate obesity-related oxidative stress and inflammation. Hence, we discuss the effects of these polyphenols on a number of signaling pathways. We note that Mediterranean diet (MedDiet) dietary polyphenols can de-regulate nicotinamide adenine dinucleotide phosphate (NADPH) oxidase (NOX) and NF-κB-mediated oxidative stress, and metabolic inflammation. MedDiet polyphenols are also effective in upregulating downstream effectors of several proteins, chiefly AMPK.

## 1. Introduction

Since being noticed in the 1960s for its beneficial role in coronary heart disease, consistent evidence shows that a Mediterranean diet (MedDiet) can prevent diet-related chronic diseases, such as metabolic syndrome including obesity [1]. MedDiet is a generic term that refers to the traditional eating habits in countries bordering the Mediterranean Sea, such as Greece and Italy. Although eating styles may vary among countries, greater intake of extra virgin olive oil, whole cereal grains, fruits, vegetables, legumes and nuts; low to moderate intake of dairy products, red meat and red wine; and low intake of sweets and eggs are the common components of MedDiet [2]. Various studies have demonstrated that Mediterranean-style diets provide high levels of phytochemicals, including dietary polyphenols [3,4,5,6], which have been reported to exert beneficial biological effects, including antioxidant, anti-inflammatory, immunomodulatory, antitumoral, antidiabetic and anti-obesity activities [4,7,8,9,10,11,12,13,14].

Overnutrition and inadequate/unbalanced nutrient consumption are major risk factors for obesity [15,16]. Obesity can be defined as an abnormal or excessive fat accumulation that presents a risk to health. Obesity is accompanied by low-grade inflammation characterized by increased pro-inflammatory cytokines and adipokines, and release of interleukin-1β (IL-1β), interleukin-6 (IL-6), TNF-α, and leptin by white adipose tissue (WAT) cells or inflammatory cells infiltrating obese adipose tissue [17,18]. Many efforts have already been undertaken to develop novel drugs targeting proteins involved in the pathogenesis of obesity, including enzymes and transcription factors with little to no side effects on cardiovascular function [19]. Hence, there has been an increasing interest in antioxidant nutraceuticals, including dietary polyphenols, due to their anti-obesity potential [7]. Dietary polyphenol-based therapeutic approaches have been gaining interest as treatment options against chronic inflammatory diseases because of their effectiveness and nontoxic nature [20,21]. A number of review articles have provided a helpful context in this regard to enhance our understanding of the role of polyphenols in obesity [7,22,23,24,25,26,27]. For example, Ramadori et al. have shown that resveratrol administration to diet-induced obese mice improved hyperinsulinemia through the modulation of hypothalamic nuclear factor-κB inflammatory signaling [28]. Likewise, resveratrol, quercetin, and Epigallocatechin-3-O-gallate (EGCG) have been reported to modulate AMPK signaling pathways, a major metabolic-sensing protein which plays a pivotal role in preventing metabolic disorders through different mechanisms. Thus, AMPK has been suggested as a potential target for obesity prevention by naturally occurring polyphenols [22,23,25]. Yet, Mohamed et al. argue the exploration of synergistic activity of polyphenols as an alternative strategy for treatment of obesity [24]. The anti-obesity effects of Mediterranean dietary polyphenols have been reviewed by Castro-Barquero et al. [26], but comprehensive reports on the effect of MedDiet on inflammation and oxidative stress associated with obesity are still lacking. In the current review, we will highlight the effect of polyphenols commonly found in a Mediterranean-style diet on oxidative stress and inflammation associated with obesity.

## 2. Nutrition Transition, Oxidative Stress and Inflammation

During the past few decades, both developed and developing countries have witnessed significant shifts in food habits. In fact, there is frequent consumption of fast food and processed, high-calorie food at the expense of ancestral traditional diets [29,30]. These shifts in food habits are known as nutrition transition characterized by high-fat (chiefly saturated animal fat), hypercaloric and salty food consumption at the expense of dietary fibers, minerals, and vitamins. Nutrition transition predisposes people to numerous noncommunicable diseases, collectively known as chronic diseases [30]. High-fat, cafeteria and Western dietary patterns have served as models for studying the impact of nutrition transition on non-communicable diseases, including obesity [31]. These diets constitute a risk factor for oxidative stress and inflammation associated with metabolic diseases [29,32,33,34,35,36,37]. It has been shown that a Western diet causes derangements of fatty acid metabolism and impairs the heart energy metabolism, mainly through oxidative phosphorylation uncoupling and free fatty acid oxidation maladaptation, concomitant with a decrease in peroxisome-proliferator-activated receptor alpha (PPARα) expression [38,39]. Moreover, a Western diet triggers toll-like receptor 4 (TLR4)-dependent increase in reactive oxygen species (ROS) production in endothelial cells associated with decreased adiponectin expression in WAT [40]. Free fatty acid (FFA)-dependent TLR4 activation can trigger c-Jun N-terminal kinase (JNK) and the major inflammatory transcription factor, NF-κB, signaling pathways in macrophages and adipocytes, which is associated with enhanced ROS production and oxidative stress [41,42]. High-fat and cafeteria-style diets can exacerbate ROS generation through nicotinamide adenine dinucleotide phosphate (NADPH) oxidase complex up-regulation in multiple tissues, including WAT [32,43]. It has been reported by us and others that high-fat and cafeteria-style diets trigger TNF-α-mediated inflammation in animal models [9,36,44,45,46]. Thus, diet-induced obesity is correlated with mitochondrial dysfunction (MD) and endoplasmic reticulum (ER) stress in WAT and liver associated with whole-body oxidative stress and the decrease in expression of antioxidant enzymes in the liver [32,33,34,35,36,40,47]. Moreover, it has been established that oxidative stress precedes the onset of high-fat diet (HFD)-induced obesity [32]. Altogether, nutrition transition may be the principal cause of whole-body oxidative stress associated with low-grade inflammation.

## 3. Oxidative Stress and Inflammation Interplay in Obesity

The etiology of obesity is multifactorial. Nevertheless, systemic oxidative stress, resulting from the impaired antioxidant defense system counteracting reactive oxygen species (ROS), is a major hallmark of obesity [17]. Indeed, superoxide anion (O_2_^•−^) overproduction during obesity can result from protein kinase C (PKC) activation, nicotinamide adenine dinucleotide phosphate (NADPH) oxidase (NOX), glyceraldehyde auto-oxidation and oxidative phosphorylation. Moreover, chronic inflammation has been demonstrated to be associated with oxidative stress in obesity [48]. Protein oxidation and protein misfolding result in adipocyte proteasomal dysfunction [49]. This latter leads to JNK hyperactivation and insulin resistance, mediated by obesity-induced endoplasmic reticulum (ER) stress in the liver [50]. Thus, ROS overproduction during mitochondrial stress is associated with exacerbated inflammation and insulin resistance in adipocytes through the activation of NF-κB [51]. NF-κB is a transcriptional factor that regulates cytokine gene expression and the inflammatory response, and it can be activated by a variety of stimuli, including dietary or endogenous lipids, hypoxia and gut-derived antigens [16]. Hence, NF-κB seems to serve as a bridge between inflammation and obesity [52]. In addition to NF-κB signaling, JNK and phospho-inositide 3-kinase (PI3-K) pathways are involved in pro-inflammatory cytokines and adipokines expression and release. The effects of inflammatory cytokines are counteracted by PPAR transcription factors, AMP-activated protein kinase (AMPK), and p38 mitogen-activated protein kinase activation, which are all regulated by adiponectin [52].

**Figure 1:**
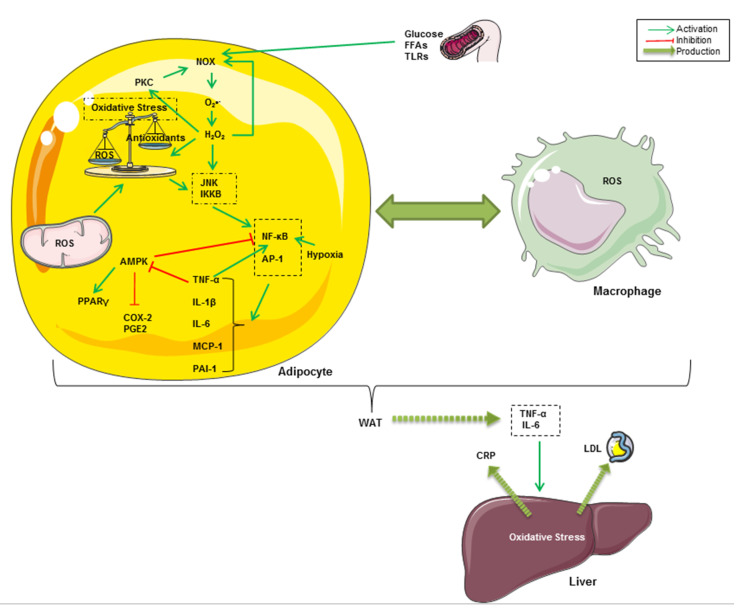
Graphical summary of some inflammatory and oxidative pathways related to obesity. High glucose intake and FFA-dependent TLR4 activation lead to the activation of NOX, which can also be activated by PKC in adipocytes. NOX mediates intracellular ROS generation, mainly O_2_^•−^, both in preadipocytes and macrophages. Likewise, mitochondrial ROS exacerbate oxidative stress and inflammatory processes in obesity. JNK mediates ROS and oxidative stress-dependent activation of NF-κB and AP-1. These latters regulate gene expression of TNF-α, IL-6, MCP-1 and PAI-1. The NF‑κB pathway might be upregulated in hypoxic adipose tissue and in response to TNF-α. Furthermore, TNF-α inhibits AMPK pathways, resulting in increased COX-2 and PGE2 and decreased PPARγ. TNF-α and IL-6 secreted from WAT enhance CRP and low-density lipoprotein (LDL) release from the liver in response to hepatic oxidative stress. A crosstalk between adipocytes and resident macrophages reinforces oxidative stress through TNF-α–mediated ROS generation within WAT cells in an autocrine and paracrine manner.

Oxidative stress occurs as a result of an imbalance between endogenous ROS production and the natural antioxidant system. As shown in Figure 1, oxidative stress is crucially implicated in the onset of chronic inflammation associated with obesity [53,54]. Indeed, it has been reported that obesogenic high-fat Western diets induce a drastic increase in oxidative stress and inflammation associated with insulin resistance and hyperglycemia [55,56,57]. In fact, high glucose intake increases ROS generation by mononuclear cells and inflammation, revealed by an increase in NF-κB and activator protein-1 (AP-1) activities in healthy human subjects [58,59]. Hyperglycemia constitutes the onset of advanced glycation end products (AGEs)-mediated oxidative stress [60]. Furthermore, the excess of nutrients such as glucose and FFAs leads to the activation of NADPH oxidase (NOX) in adipocytes [61]. NOX, which can also be activated by PKC, triggers adipogenesis in preadipocytes and mediates intracellular ROS generation, particularly of O_2_^•−^, in non-phagocytic cells [62,63,64]. PKC and NOX can, in turn, be activated by H_2_O_2_ in adipocytes [53,65]. In addition to NOX-mediated intracellular ROS production, there is evidence that the mitochondrial respiratory chain is the principal source of cellular ROS, resulting in exacerbated oxidative stress and inflammatory processes in obesity [61,66]. ROS and oxidative stress are known for the activation of stress-activated protein kinases (SAPKs, also referred to as the c-Jun N-terminal kinases, JNKs) [67]. IκB kinase β (IKKβ) has also been shown to be regulated by oxidative stress and pro-oxidants [68]. After being activated, all of these kinases trigger pro-inflammatory cytokines, chiefly through the nuclear translocation of NF-κB and AP-1 transcription factors in adipose tissue cells [69,70]. It is well known that these proteins regulate gene expression of several oxidant-responsive adipokines such as TNF-α, IL-6, monocyte chemoattractant protein-1 (MCP-1), and plasminogen activator inhibitor-1 (PAI-1) [71,72]. It has been demonstrated that IKK-dependent TNF-α activation downregulates AMPK, which plays a pivotal role in energy homeostasis and modulation of metabolic inflammation [73,74,75]. Increased AMPK phosphorylation has been correlated with enhanced anti-inflammatory, adiponectin-dependent PPARγ expression and decreased expression of cyclooxygenase 2 (COX-2) and prostaglandin E2 (PGE2) [52,76]. Therefore, it has been suggested that NF-κB-mediated inflammatory pathways in adipocytes involve the activation of COX-2 and PGE2 signaling and hypoxia associated with the decrease in anti-inflammatory, adiponectin and PPARγ expression [77,78]. TNF-α and IL-6 secreted from WAT are responsible for the onset of inflammation in other tissues and organs, such as the liver where they enhance hepatic C-reactive protein (CRP) expression [79,80,81]. Furthermore, circulating MCP-1 (also called CCL2 for chemokine C-C motif ligand 2), which is expressed and secreted from both adipocytes and activated macrophages, promotes monocyte migration and infiltration into WAT across the endothelium [81]. A crosstalk between adipocytes and resident macrophages reinforces oxidative stress through TNF-α–mediated ROS generation within WAT cells in an autocrine and paracrine manner [82]. Taken together, all of these events result in local and systemic low-grade inflammation, which can lead to multiple pathogenic outcomes ranging from type 2 diabetes to pro-oncogenic events associated with obesity [83].

## 4. Polyphenols as the Most Abundant Antioxidant in MedDiet

Evidence from human studies, generated through a cross-sectional investigation, showed that adherence to the MedDiet is inversely associated with obesity (prevalence ratio = 0.96) [84]. Indeed, a multi-center randomized trial showed that higher adherence to MedDiet is associated with decreased overweight/obesity prevalence, reflected by a lower score of body mass index [85]. Likewise, a recent study conducted on participants with abdominal obesity showed that adherence to MedDiet is associated with low peripheral glucose, total cholesterol and low-density lipoprotein (LDL) cholesterol levels [86]. As far as epigenetics is concerned, MedDiet has been suggested to exhibit positive effects on cardiovascular diseases, including obesity, through the modulation of the circadian locomotor output cycles protein kaput (CLOCK) gene, which is well-known to regulate glucose metabolism [87]. Moreover, NF-κB has been demonstrated to mediate obesity-related inflammation [88]. Interestingly, on the basis of a randomized intervention trial, it has been suggested that bioactive polyphenols in MedDiet may improve low-grade chronic inflammatory states [89].

**Figure 2:**
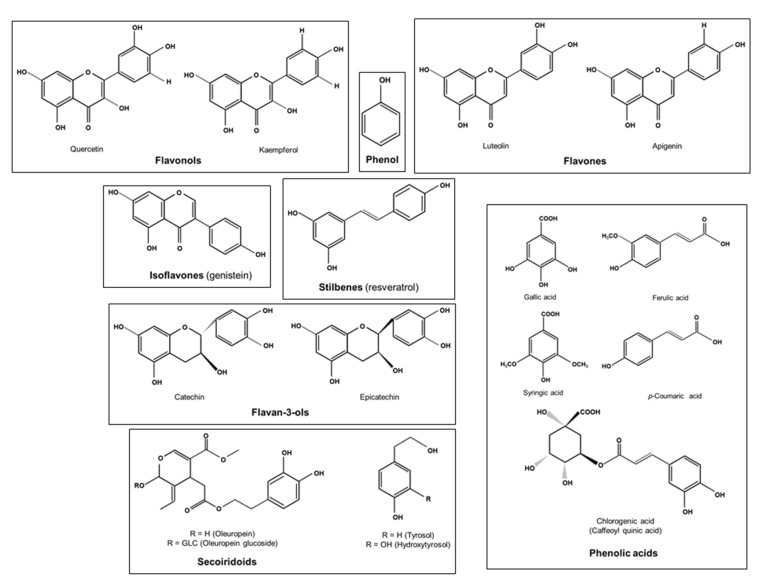
Chemical structure of some dietary polyphenols.

Phenolic compounds or polyphenols are secondary plant metabolites arising biogenetically from either the shikimate/phenylpropanoid pathway or the polyketide acetate/malonate pathway, or both [90]. With more than 8000 molecules, bioavailability and other properties of polyphenols differ from one structure to another [91]. Chemically speaking, the term phenol refers to a homologous series of compounds containing a hydroxyl group bound directly to an aromatic ring. Hence, polyphenols can be defined as substances widely distributed in the plant kingdom with more than one phenyl ring and one or more hydroxyl substituents [92]. Their structure can range from simple compounds, such as phenolic acids and stilbenes, to complex polymers derived from simple substances with high molecular mass, such as tannins (Figure 2) [93]. In the literature, there are multiple classifications of polyphenols from both edible and non-edible plants. However, dietary polyphenols are commonly categorized into two main groups: flavonoid and non-flavonoid polyphenols [94,95,96,97]. We have previously shown that polyphenols have antioxidant, anti-inflammatory, immunomodulatory, antitumor and anti-obesity properties [9,10,12,13,14].

Numerous studies have reported that polyphenols are the most abundant antioxidants in MedDiet [91,98,99,100]. Phenolic acids, such as gallic, ferulic and other hydroxycinnamic acid derivatives, are found in a variety of foods, including olive oil, whole grains, fruits, vegetables, nuts, tea, coffee and red wine [10,95,101,102]. Some phenolic acids have been reported to stimulate the secretion of adiponectin and the phosphorylation of AMPK associated with inhibition of NF-κB activation and macrophage infiltration, resulting in reduced adipogenesis and adipose inflammation in vitro and in obese animals [103,104,105]. Flavonoids, with about 6000 molecules, are the largest group of polyphenols [106]. These phenolic compounds are found in grains, fruits, vegetables, extra virgin olive oil (EVOO) and beverages such as tea, coffee and red wine [107]. Flavonoids exert prominent anti-oxidant and anti-inflammatory activities through various mechanisms. In addition to their role in food intake regulation and nutrition absorption, a growing body of evidence supports that flavonoids increase adiponectin and AMPK activation and counteract NF-κB and inducible nitric oxide synthase (iNOS) signaling pathways, resulting in reduced oxidative damage and inflammation associated with obesity [108,109,110,111]. Out of the main classes of EVOO polyphenols, hydroxytyrosol and its derivative, oleuropein, have been demonstrated to inhibit low-density lipoprotein (LDL) oxidation in vitro and contribute to management of many metabolic disorders, including obesity, through different mechanisms [112,113,114,115]. Stilbenes are a class of polyphenols that chiefly contain resveratrol, which is largely found in grape seeds and red wine. Resveratrol has been shown to be well absorbed across the gastrointestinal tract, even by obese humans [116]. It has been shown that resveratrol inhibits NADPH-induced oxidative stress in RAW 264.7 macrophages [117]. Other studies have shown that resveratrol can increase glutathione peroxidase (GPX) and superoxide dismutase (SOD) expression and activity in animal models, and it modulates gene expression of many antioxidants and anti-inflammatory molecules in obese subjects [118,119]. Common bioavailable phenolic compounds, known for their biological proprieties, in MedDiet are listed in Table 1.

## 5. MedDiet Polyphenols Counteract Oxidative Stress and Inflammation Associated with Obesity

Evidence suggests that the management of oxidative stress and inflammation may provide opportunities for the prevention and possible treatment of chronic diseases, including obesity [127]. Therefore, inflammatory and/or ROS-dependent signaling pathways are critical targets of several cardioprotective drugs and antioxidant nutraceuticals, such as polyphenols [128]. Hence, an understanding of the anti-inflammatory effects of a polyphenol-rich MedDiet is fundamental to adopt preventive and therapeutic strategies for diet-related metabolic diseases, including obesity.

It is well established that NOX-mediated oxidative stress can trigger low-grade inflammation [17]. Interestingly, polyphenols from EVOO, the usual component of MedDiet, significantly counteract pro-oxidant enzymes NOX-2 and NOX-4 and mRNA expression in adipocytes, and concomitantly reduce IL-1β and COX-2 mRNA expression and increase the expression of PPARγ mRNA. EVOO polyphenols also attenuate TNF-α-induced NF-κB activation [129]. It has been argued that excessive macronutrient-induced endothelial hyperpermeability is crucial to obesity pathogenesis. In fact, in response to cytokines such as MCP-1, endothelial cells facilitate macrophage infiltration into adipose tissue and, ultimately, enable macrophages to promote the inflammatory process [130]. Interestingly, it has been suggested that green tea polyphenol-induced NOX down-regulation contributes to a decrease in ROS production and alleviates endothelial hyperpermeability in HFD-fed rats [131]. Park et al. have demonstrated that resveratrol inhibits NOX-1 expression, concomitant with a decrease in ROS generation and MCP-1 mRNA and protein expression. Thereby, it has been suggested that resveratrol abolishes the NOX-mediated COX-2/PGE2 pathway in murine resident peritoneal macrophages [132]. In line with these findings, grape polyphenols have been reported to inhibit NOX activation and to activate PPARγ, which has been suggested to antagonize NF-κB activation, resulting in attenuated oxidative stress and inflammation associated with obesity [133]. PKC, which can be activated by saturated fatty acids (SFAs), may be an upstream regulator of NOX activity [63,133,134]. Therefore, PKC can constitute a therapeutic target for obesity management. Epigallocatechin-3-O-gallate (EGCG), the major catechin found in green tea, has been shown to attenuate inflammation in high glucose-treated endothelial cells through downregulation of PKC and NF-κB, which promotes IL-1β and MCP-1 pro-inflammatory cytokines transcription [135]. Quercetin-3-glucoside, which is another tea flavonoid that is found in onions and apples [136,137], has been reported to diminish IL-1β and MCP-1 mRNA expression in TNF-α-stimulated human adipocytes [7]. Similarly, *p*-coumaric acid, quercetin and resveratrol curtail TNF-α-induced MCP-1 production, which is concomitant with decreased PAI-1 and ROS generation in 3T3-L1 adipocytes. Furthermore, these polyphenols increase the release of adiponectin, glutathione (GSH) and anti-oxidant enzymes, including SOD, GPx and glutathione *S*-transferase (GST), in 3T3-L1 adipocytes [138].

Nuclear factor erythroid 2 (NF-E2)-related factor 2 (Nrf2) is a transcription factor that has been proposed as a therapeutic target for metabolic syndromes, including obesity, due to its mediation in triggering metabolic regulators such as PPARγ and antioxidant response element (ARE) in the liver and WAT [139]. Quercetin, which is one of the two main representative molecules of the flavonol subgroup [93], activates Nrf2 in RAW264.7 macrophages [140]. This activation of Nrf2 is accompanied by inhibition of the NF-κB pathway, and decreased mRNA expression of TNF-α, iNOS, IL-1β, IL-6 and macrophage inflammatory protein 1α (MIP1α). An in vivo study revealed that quercetin reduces the concentration of serum inflammation biomarkers (CRP and PAI-1) in obese mice [141]. Luteolin, abundantly present in MedDiet, has been found to exert anti-inflammatory activity through inhibiting NF-κB and AP-1 pathways, leading to the suppression of TNF-α, IL-6, iNOS and COX-2 gene expression in macrophages [142].

**Figure 3:**
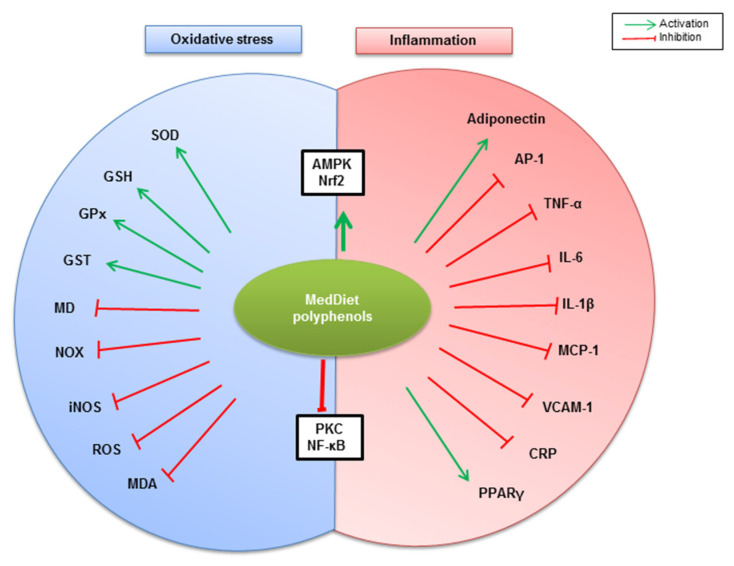
Summary of potential mechanisms by which MedDiet polyphenols modulate oxidative stress and inflammation associated with obesity. MedDiet polyphenols potentiate both AMPK- and Nrf2-mediated anti-inflammatory and antioxidant pathways, reflected by anti-inflammatory adiponectin, PPARγ, and endogenous antioxidants (SOD, GSH, GPx, and GST) upregulation. On the other hand, PKC and NF-κB-dependent inflammation and oxidative stress are counteracted by MedDiet polyphenols, reflected by the downregulation of pro-inflammatory molecules (AP-1, TNF-α, IL-6, IL-1β, MCP-1, VCAM-1, and CRP), and oxidative stress mediators and operators (MD, NOX, iNOS, ROS, MDA).

A growing body of evidence indicates that AMPK plays a pivotal role in regulating whole-body metabolism, including obesity-related metabolic-inflammation [75]. It has been reported that altered AMPK activation by phosphorylation is associated with inflammatory states in both mouse models of obesity and in obese subjects [143,144]. Indeed, it has been observed that both inflammation and oxidative stress are improved partly through adiponectin/AMPK pathways. Therefore, AMPK has been considered as an attractive therapeutic target for obesity management [20]. Ferulic acid, largely found in whole grains, has been shown to exert anti-obesity activity through the upregulation of AMPK phosphorylation, which is accompanied by a decrease in whole-body oxidative stress and inflammation—evidenced by decreased ROS generation, pro-inflammatory cytokines production, adhesion molecules expression, and circulating LDL levels—and increased adiponectin expression and circulating high-density lipoprotein (HDL) levels in HFD-fed obese mice [105]. Likewise, AMPK signaling partially mediates resveratrol-dependent anti-oxidant effects through reversing mitochondrial dysfunction, increasing total antioxidative capability, elevating activity of SOD and GPx and curtailing malondialdehyde (MDA) and carbonyl protein contents in HFD-fed obese mice [145]. Consistent with these observations, resveratrol inhibits NF-κB activation, resulting in reduced TNF-α, IL-1β, IL-6, and COX-2 mRNA expression, and reduced IL-6 and PGE2 secretion in 3T3-L1-derived adipocytes [146]. Hydroxytyrosol has been reported to modulate oxidative stress and inflammation associated with obesity. Indeed, hydroxytyrosol increases AMPK activation, Nrf2 and PPARγ mRNA expression, and adiponectin expression in adipocytes, and decreases PGE2 expression, VCAM-1 vascular cell adhesion protein expression and circulating CRP inflammatory marker [3]. Altogether, MedDiet’s phenolic compounds can modulate oxidative stress and inflammation associated with obesity through different mechanisms that are most likely orchestrated by NOX, AMPK, NF-κB, PKC and Nrf2 signaling (Figure 3).

## 6. Concluding Remarks

A Mediterranean-style dietary pattern is effective in managing nutrition transition-related metabolic disorders, including obesity. Among the antioxidant nutraceuticals of MedDiet, polyphenols exhibit antioxidant and anti-inflammatory properties. Hydroxycinnamic acids, flavonoids (chiefly quercetin and catechins), resveratrol, oleuropein and hydroxytyrosol are the most studied phenolic compounds. MedDiet polyphenols alleviate inflammation and oxidative stress in obesity, in part, through the regulation of AMPK and NF-kB signaling pathways. Hence, targeting these pathways may be a prominent therapeutic approach for managing obesity.

## Figures and Tables

**Table 1:** Polyphenols commonly found in a Mediterranean-style diet.

Polyphenols		Principal Dietary Sources
**Flavonoids**	**Flavonols** (quercetin, Kaempferol)	Onions, apples, berries, tea, beans, tomatoes, grapes, medicinal plants such as *Osyris alba* root bark, spices such as coriander seeds [93,120,121]
	**Flavones** (apigenin, luteolin)	Black olives, olive oil, wheat grains, fruits, vegetables [91,93]
	**Isoflavones** (genistein)	Bread [122]
	**Anthocyanidins** (anthocyanins)	Maize, strawberries, blood oranges, pomegranates, beans, red onions [93,105]
	**Flavanols** (catechins, epicatechin)	Tea, grapes, apples, nuts such as almonds and pistachos, red wine [93]
**Non-flavonoids**	**Phenolic acids** (gallic, ferulic, *p*-coumaric, caffeic syringic, and chlorogenic acid)	Whole cereal grains, tea, carob leaves, *Osyris alba* root bark, garlic, spices such as coriander seeds, black cumin seeds, fenugreek seeds [10,14,93,105,120,121,123,124]
	**Stilbenes** (resveratrol)	Grapes, peanuts, plums, beans, red wine [93,125]
	**Secoiridoids** (oleuropein, hydroxytyrosol)	Olive oil [122,126]
	**Lignans**	Whole-grain cereals, olive oil [93,126]

## Abbreviations

AMPK	AMP-activated protein kinase
AP-1	activator protein-1
ARE	Antioxidant Response Element
COX-2	cyclooxygenase 2
CRP	C-reactive protein
EGCG	Epigallocatechin-3-O-gallate
ER	Endoplasmic Reticulum
EVOO	extra virgin olive oil
FFAs	free fatty acids
GPx	glutathione peroxidase
GSH	glutathione
GST	glutathione *S*-transferase
HDL	high-density lipoprotein
HFD	high fat diet
IKK	IκB kinase
IL	interleukin
iNOS	inducible nitric oxide synthase
IκB	Inhibitor of κB
JNK	c-Jun N-terminal kinase
LDL	low-density lipoprotein
MCP-1	monocyte chemoattractant protein-1
MD	mitochondrial dysfunction
MDA	malondialdehyde
MedDiet	Mediterranean diet
MIP1α	macrophage inflammatory protein 1α
NF-κB	nuclear factor-kappa B
NOX	nicotinamide adenine dinucleotide phosphate (NADPH) oxidase
Nrf2	Nuclear factor erythroid 2 (NF-E2)-related factor 2
PAI-1	plasminogen activator inhibitor-1
PGE2	prostaglandin E2
PI3-K	phospho-inositide 3-kinase
PKC	protein kinase C
PPAR	peroxisome proliferator-activated receptor
ROS	reactive oxygen species
SAPKs	Stress-Activated Protein Kinases
SFAs	saturated fatty acids
SOD	superoxide dismutase
TLR	Toll-like receptor
TNF-α	tumor necrosis factor-alpha
VCAM-1	vascular cell adhesion molecule-1
WAT	white adipose tissue.
